# ‘Let’s Talk About Children’ family focused practice for children of parents with schizophrenia and bipolar disorder: protocol for a randomized controlled trial

**DOI:** 10.1186/s12888-023-05457-6

**Published:** 2024-01-02

**Authors:** Lingzi Xu, Zhi Sheng, Tianhang Zhou, Chenmei Xie, Xun Wang, Wufang Zhang, Tingfang Wu, Petra Gronholm, Dafang Chen, Hong Ma, Graham Thornicroft, Lili Guan, Xin Yu

**Affiliations:** 1 Peking University Sixth Hospital, Peking University Institute of Mental Health, Beijing, China; 2 Health Service and Population Research Department, Institute of Psychiatry, Psychology and Neuroscience, King’s College London, London, UK; 3 Department of Epidemiology and Biostatistics, School of Public Health, Peking University, Beijing, China; 4 Centre for Global Mental Health and Centre for Implementation Science, Health Service and Population Research Department, Institute of Psychiatry, Psychology and Neuroscience, King’s College London, London, UK

**Keywords:** Bipolar disorder, Children of parents with mental illness, Family focused practice, Family therapy, Parental mental illness, Schizophrenia, Severe mental disorder

## Abstract

**Introduction:**

‘Let’s Talk About Children’ is a brief family focused intervention developed to improve mental health outcomes of children of parents with mental illness (COPMI). This study aims to assess the efficacy of LTC in improving mental health of children of parents with schizophrenia or bipolar disorder in China.

**Methods:**

The planned study is a multicentre parallel group randomized wait-list controlled trial. A total of 400 eligible families with children aged 8 to 18 years will be recruited, 200 each for families with parental schizophrenia or bipolar disorder. The intervention group will receive Let’s Talk About Children delivered by a trained therapist, while the control group will receive treatment as usual. The primary outcomes are child mental health measured by the strengths and difficulties questionnaire and parent–child communication measured using the parent-adolescent communication scale. Parental mental health and family functioning are secondary outcomes. This study also plans to explore mediating factors for the effect of Let’s Talk About Children on child mental health, as well as conduct a cost-effectiveness analysis on using Let’s Talk About Children in China.

**Conclusion:**

The present study will provide evidence for the efficacy of Let’s Talk About Children in families with parental schizophrenia and bipolar disorder in China. In addition, it will evaluate potential mechanisms of action and cost-effectiveness of Let’s Talk About Children, providing a basis for future implementation.

**Trial registration:**

ChiCTR2300073904.

## Introduction

Up to one in five children and adolescents worldwide are growing up in families where at least one parent experiences mental illness [1]. Children of parents with mental illness (COPMI) not only have a higher genetic risk for the condition, but on average also experience more childhood adverse events including emotional abuse, domestic violence and bullying [27]. Studies also show that mental illnesses amplify parenting challenges, may interfere with parent–child bonding, and can lead to children to taking on household burdens incongruous with their developmental age [6, 7, 13, 14, 18]. These factors impair the family’s ability to nurture child growth and may contribute to the transgenerational transmission of mental health conditions. Children whose parents experience severe mental disorders such as schizophrenia or bipolar disorder are especially disadvantaged and are at greater risk of having poorer outcomes in adulthood than children of parents without a mental health condition. Across multiple studies, the proportion of children with familial high risk who develop schizophrenia in adulthood ranges from 12.3% to up to 18.8% [11, 13, 21], compared to less than 1% in the general population [16].

Research has been conducted to identify modifiable risk factors that may potentially prevent or attenuate symptoms of mental disorders in offspring of parents with mental illness. Poor parenting behaviour and family functioning have been found to mediate the associations between parental mental disorder and offspring mental health outcomes [3]. Lack of appropriate information and support may lead to COPMI feeling guilty for causing their parent’s illness, which can in turn negatively affects their own emotions and behaviours [26]. These findings suggest that improving parenting capabilities and increased communication about parental mental health conditions may potentially improve outcomes for COPMI. However, many parents with mental illnesses lack parenting support tailored to their needs and also do not have the confidence to talk about their conditions with their children [19, 20].

In response to this issue, several family focused practices have been developed to support parents with mental health conditions and subsequently improve the mental well-being of COPMI [28]. One of these interventions is Let’s Talk About Children (LTC). LTC is a brief program designed to be delivered in low resource settings to help parents with mental illnesses build protective relationships with their children and improve family functioning [1, 2]. Evidence shows that LTC decreases depression and anxiety and increases prosocial behaviour and health related quality of life in COPMI [10, 24]. LTC also improves parental motivation to engage with mental health treatment, and increases their self-acceptance and parenting confidence [26]. These positive effects may potentially prevent future mental illness in COPMI as well as increase resilience if mental health issues may occur.

Previous applications of LTC have been largely in families where parent(s) suffer from depression [10, 24]. We found only one study on the efficacy of LTC in families with parental bipolar disorder [28] and none in families with parental schizophrenia, despite the greater disadvantage faced by children in these circumstances. Moreover, LTC has been implemented in various countries including Finland [24], Greece [10], Japan [26], Australia [19] and the USA [20], however, it has not yet been translated into Chinese. Mental health services in China generally focus on the treatment of patients with clinically diagnosed conditions. There is a lack of evidence-based therapies that can be used in children at high risk or exhibiting subthreshold symptoms of mental disorders in China. Our team has previously translated and adapted LTC for use in China. A pilot feasibility study of 30 COPMI families found that LTC increased parenting confidence and improved parenting satisfaction in 60% and 90% of participating families respectively (unpublished data). This study aims to bridge this knowledge gap by conducting a randomized controlled trial (RCT) to investigate the effects of LTC in families with parental schizophrenia or bipolar disorder in China.

## Objectives

The primary objective of this study is to assess the efficacy of LTC in improving mental health of children of parents with schizophrenia or bipolar disorder in China. We hypothesize that the 3-session LTC intervention will result in improvements in child mental health or parent–child communication compared to a wait list control group.

**Fig. 1:**
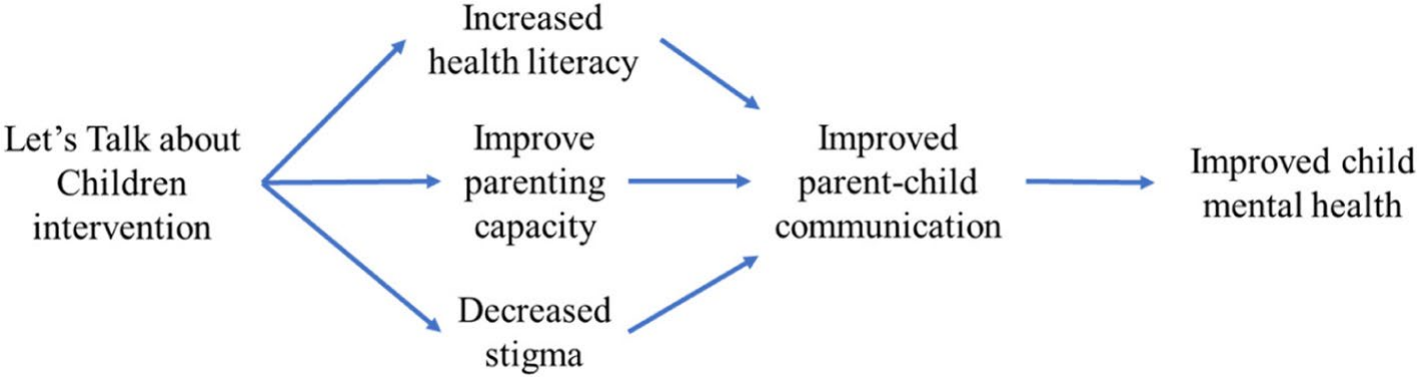
Hypothesized mechanisms of action for improvement of child mental health using ‘Let’s Talk about Children’

The secondary objectives are to:

1. Assess the efficacy of LTC in improving the mental health of parents experiencing schizophrenia or bipolar disorder.
2. Explore potential mechanisms of action for improvement of child mental health using LTC. A proposed causal pathway is presented in Fig. 1.
3. Assess the cost effectiveness of LTC in families with parental schizophrenia or bipolar disorder in China.

## Methods

### Design

**Fig. 2:**
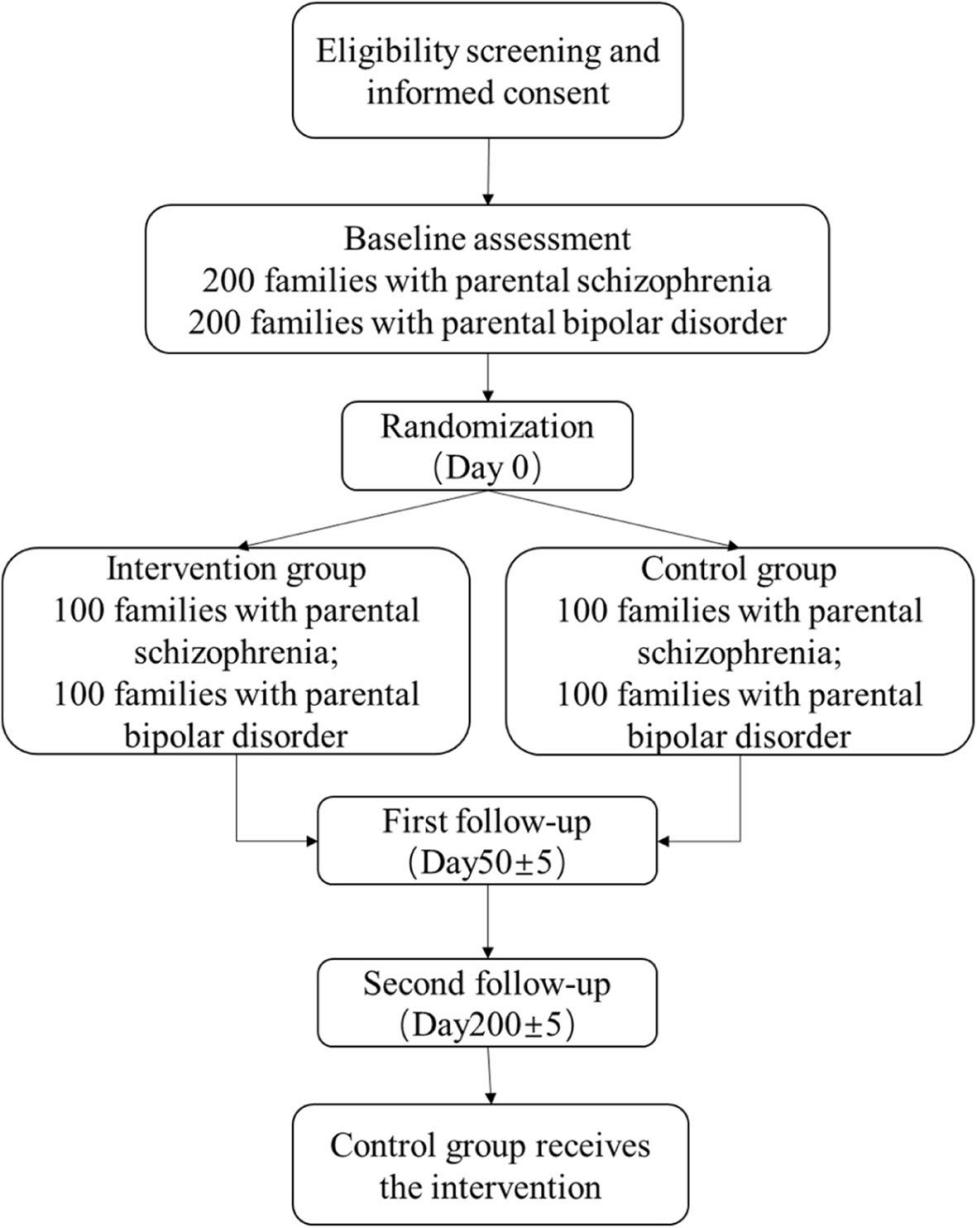
Study design

A flowchart of the study design is shown in Fig. 2. The WHO Trial Registration Data Set is provided in Table 1. The planned study is a multicentre parallel group randomized wait-list controlled trial. A total of 400 eligible families with children aged 8 (inclusive) to 18 (not inclusive) years will be identified and recruited, 200 each for families with parental schizophrenia or bipolar disorder. The study was approved by the Ethics Committee of Peking University Institute of Mental Health, reference number 2023(51). This trial is registered in the Chinese Clinical Trial Registry (ChiCTR), ChiCTR2300073904. This study was initiated by LLG and is funded through a research grant from the China Medical Board (grant #21–435). The coordinating centre (Peking University Institute of Mental Health) will conduct intervention training, random sequence generation, data management, quality control, audit trial conduct and supervise intervention implementation. This protocol is reported in accordance with Spirit guidelines [5].

**Table 1:** Summary of study design

Data category	Information
Primary registry and trial identifying number	ChiCTR ChiCTR2300073904
Date of registration in primary registry	2023–07-25
Secondary identifying numbers	Protocol number CAFF20230725
Source(s) of monetary or material support	China Medical Board
Primary sponsor	China Medical Board
Secondary sponsor(s)	Care for Families Program (Beijing, China)
Contact for public queries	Lili Guan, MD, MPH, guanlili@bjmu.edu.cn
Contact for scientific queries	Lili Guan, MD, MPH, guanlili@bjmu.edu.cn
Public title	"Let’s Talk About Children" Family Focused Intervention for Children of Parents with Severe Mental Disorders: Protocol for a Randomized Controlled Trial
Scientific title	"Let’s Talk About Children" Family Focused Intervention for Children of Parents with Severe Mental Disorders: Protocol for a Randomized Controlled Trial
Countries of recruitment	China
Health condition(s) or problem(s) studied	Schizophrenia, Bipolar Disorder
Intervention(s)	Intervention group: Let’s Talk about Children; Control group: Treatment as usual, which typically includes medication, case management, outpatient consultations and rehabilitation services;
Key inclusion and exclusion criteria	Inclusion criteria are: 1. At least one child that is at least 8 years old and not yet 18 years old in the family; 2. One biological parent has met ICD-10 diagnostic criteria for schizophrenia or bipolar disorder for at least one year; 3. In the past year, the patient has lived with the child(ren) for at least 6 months; 4. Patient and their child(ren) can understand the study and provide written informed consent Exclusion criteria are: 1. Previous or current participation in family therapy; 2. Patient has been hospitalized or has visited the emergency room in the past month due to their mental illness, or presence of self-harm, suicidal or aggressive behaviour in the past month; 3. Patient or their child(ren) has severe cognitive impairment, learning disabilities, communication difficulties or behavioural problems; 4. Anticipated difficulty in completing the intervention or follow-ups for the next 7 months for any reason (i.e., moving away); 5. Single parent households at the time of enrolment (parent is divorced or widow(er)ed and is not remarried); 6. Both parents suffer from severe mental illnesses, including schizophrenia, bipolar disorder, schizoaffective disorder, delusional disorder, or other psychotic disorders Discontinuation criteria: 1. During the intervention or follow-up period, patient is hospitalized or visits the emergency room due to their mental illness, or exhibits self-harm, suicidal or aggressive behaviour, or is diagnosed with other severe medical conditions; 2. During the intervention or follow-up period, the spouse or child(ren) is diagnosed with a severe mental disorder (including schizophrenia, bipolar disorder, schizoaffective disorder, delusional disorder, or other psychotic disorders), or other severe medical conditions
Study type	Randomized Clinical Trial
Date of first enrolment	September, 2023
Target sample size	400
Recruitment status	Ongoing
Primary outcome(s)	Strengths and Difficulties Questionnaire; Parent-Adolescent Communication Scale;
Key secondary outcomes	Child mental health; Child self-esteem; Quality of life; Parenting; Mental health knowledge

### Setting

Participants will be recruited from 10 district mental health centres across China. Most of the centres participating in this study have already provided services to or completed research on COPMI through a previous epidemiological survey initiated by our group (results unpublished). Study centres all provide community mental health services to patients with severe mental disorders who serve as index patients to identify target families. Psychiatrists or community mental health professionals in these centres will conduct recruitment, enrolment, data collection and research coordination.

### Participants

This study will recruit families with children being raised by parents with schizophrenia or bipolar disorder. Written informed consent will be required from the patient, the patient’s spouse and the child(ren) before enrolment. When the primary caregiver of the child is not a parent, informed consent will also be acquired from this primary caregiver. The patient, the patient’s spouse and/or the primary caregiver will be introduced to the study in a face-to-face interview by researchers and provided with an electronic link to the full consent form. Upon parental consent for the participation of their child(ren), they will be instructed to inform their child(ren) of the study and have their child(ren) read and sign an informed consent form that discloses participation in a family therapy intervention study. Participants are made aware of their right to withdraw at any time from the study without compromising their routine care.

### Intervention

LTC is a brief family focused intervention design to be delivered with minimal training [2]. The LTC intervention has been previously translated into Chinese and pilot tested by our study group (unpublished data). Therapists with prior experience working with patients with severe mental illnesses will be recruited to receive LTC training provided by our study team. Remote and on-site supervision will be conducted routinely to ensure treatment quality. The intervention will be delivered to the families in three sessions across one month, of which the first is a preparatory session, with at least one week between session 2 and session 3. Each session will last around 60 min. Therapeutic goals of each session are summarized in Table 2.

**Table 2:** Summary of therapeutic goals in each session of ‘Let’s Talk About Children’

Session	Goals
Session 1 (Preparation)	Introduce the intervention. Explore potential concerns about the intervention. Set up future meetings
Session 2	Explore strengths and weaknesses of the child(ren) and concerns about parenting. Each child will be discussed separately. Length of this session may be extended if needed
Session 3	Review the previous session. Discuss impacts of mental illness on parenting. Explore ways parents can help build on strengths and support weaknesses of the child(ren)

### Control

This study employs a wait list control design. The control group will receive treatment as usual, which typically includes medication, case management, outpatient consultations and/or rehabilitation services for the patients. Once the study is complete, families in the control group will be offered free LTC therapy.

### Randomization

Families will be stratified by study centre and diagnosis. Random number sequences will be generated in a 1:1 ratio using statistical software by an independent statistician and written into the electronic data collection (EDC) software developed specifically for this study. The random number sequence will be generated by third-party engineers commissioned to develop the EDC software and cannot be accessed by the research team. Participants will be allocated automatically within the software after completing screening assessments and enrolment is confirmed.

This study will not use masking. Because this study employs wait list control, participants and therapists will be aware of allocation status. Masking of researchers will also be difficult as in most study sites, researchers share office space with therapists, therefore they will likely see participants coming in for sessions.

### Follow up schedule

Data will be collected at baseline, 50 ± 5 and 200 ± 5 days after enrolment (around 1 and 6 months after the final LTC session). We chose to wait one month after the final session before collecting data to ensure subjects have time to apply skills and insights they gained from the intervention at home. Study staff will record demographic information during enrolment interviews. Patients, their spouses and their child(ren) will all be asked to complete questionnaires at baseline and follow-ups (see Table 3 for detailed schedule). When the primary caregiver of the child is not a parent, this primary caregiver will also be asked to complete the questionnaire.

**Table 3:**
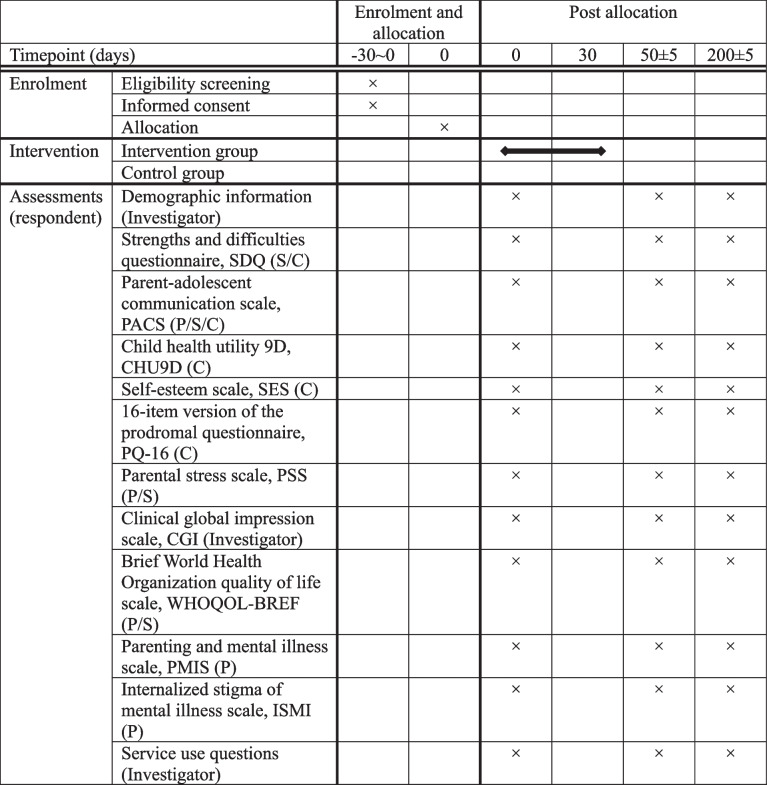
Schedule of enrolment, interventions, and assessments

*Abbreviations*: *P* parent, *S* spouse, *C* child

### Data collection and outcome measures

Data collection will be conducted using software designed specifically for this study. Patients, spouses, children, and/or caregivers will complete assessments independently under the assistance of researchers.

This study has two primary outcomes, child mental health and parent–child communication. Child mental health will be measured using the strengths and difficulties questionnaire (SDQ) [12], a widely used measure with 25 items covering emotional symptoms, conduct problems, hyperactivity/inattention, peer relationship problems and prosocial behaviour. Parent–child communication will be measured using the parent-adolescent communication scale (PACS) [30], which has been previously used in children aged 7 and above [9] and comprises two subscales, open communication and communication problems, each with 10 questions. A significant improvement of either scale compared to the control group will be considered a positive result.

Secondary objectives will be assessed using the following measures:

1. The 16-item version of the prodromal questionnaire (PQ-16) [15] will be used to assess the prodromal state in the child(ren). Child self-esteem will be assessed using the self-esteem scale (SES) [23].
2. The clinical global impression scale (CGI) will be assessed by mental health professionals to reflect potential improvements in patient mental health.
3. Parenting is a hypothesized mediating factors for the effect of LTC on parent–child communication. Parenting ability and stress will be measured using the parenting and mental illness scale (PMIS) [8] and the parental stress scale (PSS) [4], respectively.
4. The final secondary objective of this study is to assess the cost effectiveness of LTC in China. To quantify utility, we will measure quality of life using the child health utility 9D (CHU9D) [25] in children and the brief World Health Organization quality of life scale (WHOQOL-BREF) [29] in patients.
5. To provide preliminary information for future implementation of LTC, we designed 5 simple questions regarding current use of mental health services in patients.

### Sample size calculations

Sample size was calculated using SDQ as the primary outcome. Data from multiple countries (https://www.sdqinfo.org/g0.html) show that the population standard deviation of SDQ is roughly 5 points. Considering an SDQ difference of 3 points to be a clinically significant difference, at α = 0.05 and β = 0.9 levels, assuming a two-sided hypothesis and using 1:1 ratio of random allocation, the sample size needed for analysis is 120. As families with schizophrenia and bipolar disorder will be analysed separately, assuming a 40% dropout rate, 200 subjects will be needed for each diagnostic category. Therefore, 200 families with schizophrenia, and 200 with bipolar disorder will be enrolled.

### Statistical analysis

Baseline categorical variables will be summarized as proportion of subjects with the count divided by the total number of evaluated subjects. Baseline continuous variables will be summarized as mean with standard deviation in case of normal distribution and as median with interquartile range in case of non-normal distribution. Efficacy will be assessed in the intention-to-treat population. Missing data will be imputed using the last observation carried forward method. Outcome variables will be differences between pre- and post-test scores at 50 days and 200 days.

Primary outcomes will be compared using t-tests if results conform to a normal distribution and nonparametric tests if they do not. Multivariate generalized linear regression models will be performed with outcome variables as the dependent variable, group allocation as the independent variable, and baseline variables as covariates to account for potential confounding. Data will be analysed as a whole and also separately in the two diagnosis groups. Further subgroups will be explored using interaction tests in the multivariate regression models. Potential subgroup identifiers include but are not limited to child mental health at baseline, parental disease severity, family socioeconomic status and child age. All inferences will be made at a significance level of α = 0.05.

### Data management and security

An electronic data collection software was developed specifically for this study. Researchers and therapists will have password protected accounts with different access permissions according to their role. Only the trial management group can view data from all centres. Participants will be provided one-time links to online questionnaires for their own record. The trial management group will monitor the dataset weekly for missing data, outliers, protocol deviation and other potential issues. Dropouts will be directly recorded by site staff through the EDC software. After completion of the study, de-identified data will be exported from the software by the primary statistician and stored on a password protected computer. No interim analyses are planned.

### Monitoring and auditing

A trial management group led by LG has been set up to oversee study implementation through weekly meetings and in person or online communications as needed. A biweekly video conference will be held with researchers and therapists from all study sites to communicate study progress and discuss potential issues. Members of the trial management group will visit each site periodically to audit treatment fidelity and data collection.

### Safety assessment

Adverse events will be monitored and recorded throughout the study. Few adverse events are anticipated as the intervention is clinical therapy. In case of distress, agitation or risk to self or others, subjects will be referred to psychiatric care.

### Dissemination policy

Results of this trial will be published in a peer-reviewed journal. Study findings will be disseminated through international and domestic academic meetings. A simplified version of the results will be published in our study team’s WeChat public account titled “CAFF花园” which targets COPMI, their families, mental health professionals and other people interested in the mental health of COPMI.

## Discussion

To our knowledge, this study is the first to assess LTC in families with parental schizophrenia and is the second in families with parental bipolar disorder worldwide. LTC is a brief and simple intervention that has potential to improve mental health and prevent mental disorders in high-risk children. However, LTC has only been tested in limited settings. Previous evidence regarding LTC focuses on families with parental depression [10, 24, 28]. Families with severe parental mental disorders face significantly greater parenting challenges compared to families with parental depression. Parents with severe mental disorders may exhibit violent behaviours, be absent due to hospitalization, or have poorer overall functioning. Severe mental disorders are also more stigmatized than depression [17, 22]. Therefore, families with severe parental mental disorders are in greater need of parenting support. Anecdotal reports of clinicians’ implementation of LTC in families with severe parental mental disorders have been positive [1], however there is a lack of quantitative evidence regarding its efficacy in this population. Therefore, our study will provide valuable information for future clinical practice in families with severe mental disorders worldwide.

Previous research has found that both mothers and fathers with mental illness in China have parenting concerns related to their mental illness [6, 7], however there are currently no evidence-based family focused therapies to address this issue in China. If positive, results from this study will provide much needed treatment option in Chinese speaking families with parental mental illness.

The primary limitation of this study is the inability to blind participants, therapists and research workers to randomization status, which may lead to overestimation of intervention effects because of observer bias. Another limitation is that the therapists participating in this study have no previous experience delivering LTC as this intervention was introduced to China only recently by our study team, which may result in poorer efficacy compared to experienced therapists. Our team will conduct detailed training and supervision throughout the study to ensure fidelity and congruency across different sites.

The results of this study will increase evidence for using LTC in prevention and early intervention of mental disorders in COPMI. If results are positive, further cost effectiveness analyses will be conducted which may provide a basis for widespread implementation of LTC in families with schizophrenia or bipolar disorder in China.

## Data Availability

No datasets were generated or analysed during the current study.
